# Expression changes of ER, PR, HER2, and Ki-67 in primary and metastatic breast cancer and its clinical significance

**DOI:** 10.3389/fonc.2023.1053125

**Published:** 2023-04-28

**Authors:** Xueyang Hu, Wenjun Chen, Fanfan Li, Pengfei Ren, Hongyang Wu, Congjun Zhang, Kangsheng Gu

**Affiliations:** ^1^ Department of Medical Oncology, The First Affiliated Hospital of Anhui Medical University, Hefei, China; ^2^ Phase I Clinical Center, Anhui Chest Hospital, Hefei, China; ^3^ Department of Medical Oncology, The Second Affiliated Hospital of Anhui Medical University, Hefei, China

**Keywords:** breast tumor, tumor metastasis, heterogeneity, prognosis, DFS = disease-free survival

## Abstract

**Objective:**

To explore the altered expression of estrogen receptor (ER), progesterone receptor (PR), human epidermal growth factor receptor 2 (HER2), and cell proliferation index (Ki-67) in primary and metastatic breast cancer lesions and the correlation between the primary tumor size, lymph node metastasis, Tumor Node Metastasis (TNM) stage, molecular typing, and disease-free survival (DFS) and their clinical significance.

**Methods:**

A retrospective analysis was conducted on the clinical data of 130 patients with metastatic breast cancer biopsy admitted to the Cancer Center of the Second Affiliated Hospital of Anhui Medical University in Hefei, China, from 2014–2019. The altered expression of ER, PR, HER2, and Ki-67 in primary and metastatic lesions of breast cancer was analyzed with respect to the site of metastasis, size of the primary tumor, lymph node metastasis, disease progression, and prognosis.

**Results:**

The inconsistent expression rates of ER, PR, HER2, and Ki-67 in primary and metastatic lesions were 47.69%, 51.54%, 28.10%, and 29.23%, respectively. The size of the primary lesion was not, but that accompanied by lymph node metastasis was related to the altered receptor expression. Patients with positive ER and PR expression in both primary and metastatic lesions had the longest DFS, while those with negative expression had the shortest DFS. Also, changes in HER2 expression in primary and metastatic lesions were not associated with DFS. Patients with low expression of Ki-67 in both primary and metastatic lesions had the longest DFS, while patients with high expression had the shortest DFS.

**Conclusion:**

Heterogeneity was detected in the expression levels of ER, PR, HER2, and Ki-67 in the primary and metastatic breast cancer lesions, which has a guiding significance for the treatment and prognosis of patients.

## 1 Introduction

Breast cancer remains the leading cause of cancer morbidity and mortality among women worldwide, according to the Global Cancer Statistics 2020 report (1). About 5-10% of women with breast cancer exhibit metastasis at the time of diagnosis, with a 5-year survival rate of about 25%, and 20-30% of women with early breast cancer who receive adjuvant therapy will develop metastatic breast cancer (2). Bone, lung, and pleura are the most common sites of breast cancer metastasis, and the median survival after metastasis is 0.5-3 years (3, 4). Breast cancer metastasis is usually incurable, and its molecular typing underlies clinical treatment planning. The molecular typing of breast cancer is based on immunohistochemical indicators estrogen receptor (ER), progesterone receptor (PR), human epidermal growth factor receptor 2 (HER2), and cell proliferation index (Ki-67). The expression of receptors in the recurrence and metastasis foci may change after the recurrence and metastasis of breast cancer. However, the formulation of treatment plans for many breast cancer patients is based on the expression of receptors in the primary foci, which might affect the follow-up efficacy and prognosis of patients (5). In this study, the correlation in ER, PR, HER2, and Ki-67 expression was analyzed between the primary and metastatic lesions of breast cancer and the site of metastasis, the size of the primary tumor, lymph node metastasis, tumor node metastasis (TNM) stage, disease progression, and patient prognosis. To provide a reference for the individualized follow-up treatment and prognosis evaluation of patients with breast cancer recurrence and metastasis.

## 2 Data and methods

### 2.1 Subjects

The clinicopathological data of patients who underwent breast cancer recurrence/metastasis biopsy in the Tumor Center of the Second Affiliated Hospital of Anhui Medical University in Hefei, China, from January 2014 to December 2019, were collected. Pathological specimens in this study were reviewed by two professional pathologists repeatedly for diagnosis. The characteristics included the patient’s age at diagnosis, the interval from the first diagnosis to recurrence/metastasis, site of metastasis, size of the primary tumor, whether the primary tumor has lymph node metastasis, expression of ER, PR, HER2, and Ki-67 in primary and recurrence/metastasis lesions, and disease outcome. For patients with multiple biopsy results of metastatic lesions, the first biopsy results were selected for analysis. Also, the first biopsy results of recurrent lesions were selected for analysis for patients with multiple recurrence results.

### 2.2 Definition of metastasis

Metastasis includes local recurrence and distant metastasis. Local recurrence included ipsilateral breast, ipsilateral chest wall, and ipsilateral regional lymph node metastasis. In contrast, distant metastasis included contralateral breast, contralateral chest wall, contralateral lymph node, bilateral supraclavicular lymph node, bone, and visceral metastasis.

### 2.3 Judgment of vital data

ER and PR were determined according to the immunohistochemical results. ER-positive cells ≥1% were considered ER-positive, and PR-positive cells ≥1% were considered PR-positive. HER2 (-) and (+) are negative, HER2 (+++) is positive, and HER2 (++) needs further detection by fluorescence *in situ* hybridization (FISH); HER2 is positive if *HER2* gene is amplified, otherwise negative. Ki-67<14% was considered a low expression, and Ki-67≥14% was considered a high expression. TNM staging was based on American Joint Committee on Cancer (AJCC) eighth Edition 2017. DFS was defined as the time between breast cancer diagnosis and the first recurrence/metastasis.

### 2.4 Data statistics

SPSS 23.0 software was used for statistical analysis. A paired χ^2^ test was used to analyze the expression of various immunohistochemical indexes in primary and metastatic lesions. Kaplan-Meier survival analysis was performed for disease-free survival (DFS), and logistic multifactor regression was used to analyze the effect of the treatment plan on the heterogeneity of receptor expression. p-value <0.05 indicated a statistically significant difference.

## 3 Results

### 3.1 Clinicopathological features

A total of 130, 130, 121, and 130 patients had complete ER, PR, HER2, and Ki-67 data for primary and metastatic lesions, respectively. All patients were females, with a median age of 55(range: 24-86 years) at stage of recurrence/metastasis diagnosis. All patients were pathologically diagnosed with invasive cancer, including 58 (44.62%) cases of local recurrence and 72 (55.38%) cases of distant metastasis. Distant metastasis included liver metastasis in 20 cases, lung metastasis in 17 cases, bone metastasis in 18 cases, and metastasis to other sites in 17 cases. Among them, 106 cases had complete TNM staging records, 5 received neoadjuvant therapy, and 19 presented incomplete TNM staging information. The median DFS was 50 months (**Table 1**).

**Table 1:** Patient and tumor characteristics at primary diagnosis and recurrence/metastasis.

Characteristic	Primary	Recurrence/Metastasis
Age	50.2(22-81)	55(24-86)
Tumor size(cm)	2(1-5.5)	NA
Stage at diagnosis, n(%)		NA
I	15(11.5)	
II	56(43.1)	
III	28(21.5)	
IV	12(9.2)	
Unknown	19(14.6)	
Pathology, n(%)		
Ductal	105(80.8)	110(84.6)
Lobular	10(7.7)	10(7.7)
Other	15(11.5)	10(7.7)
Therapy, n(%)		
Targeted therapy	17(13.1)	17(13.1)
Endocrine therapy	60(46.2)	35(26.9)
Taxane-containing chemotherapy	48(36.9)	40(30.7)
Anthracycline-containing chemotherapy	45(34.6)	35(26.9)
Locoregional recurrence, n(%)	NA	58(44.6)
Local		45(34.6)
Lymph node		13(10.0)
Distanst metastasis, n(%)	NA	72(55.4)
Liver		20(15.4)
Lung		17(13.1)
Bone		18(13.8)
other		17(13.1)

### 3.2 Heterogeneity of receptor expression in primary and metastatic lesions

The rate of positive staining for ER, PR, HER2 in primary lesions was 63.08%, 60.00%, 32.23%, respectively, while that in metastatic lesions was 63.08%, 48.46%, 43.80%, respectively. The rate of high expression of Ki-67 was 77.69% in primary lesions, while that in metastatic lesions was 77.69%. On the other hand, the rate of inconsistent expression for ER, PR, HER2, and Ki-67 was 47.69%, 51.54%, 28.10%, and 29.23%, respectively, between primary and metastatic lesions.

#### 3.2.1 Expression heterogeneity of ER between primary and metastatic breast cancer lesions

ER expression was inconsistent between the primary and the recurrence/metastasis lesions in 62 cases, of which 31 (23.85%) cases changed from positive to negative, and the remaining 31 (23.85%) changed from negative to positive. The primary lesion was ER-negative in 48 cases and changed to ER-positive in 31 cases (64.58%). The primary lesion was ER-positive in 82 cases and changed to ER-negative in 31 cases (37.80%). Among the patients with local recurrence, ER-positive turned negative in 17 cases, and negative turned positive in 10 cases, with an inconsistent expression rate of 46.55%. Among the patients with distant metastasis, ER-positive turned negative in 21 cases, and negative turned positive in 19 cases, with an inconsistent expression rate of 55.56% (**Table 2**).

**Table 2:** Expression of immunohistochemical indexes in primary and recurrent metastatic lesions [n (%)].

		Primary lesions								
			ER		PR		HER2		Ki-67	
			Positive	Negative	Positive	Negative	Positive	Negative	High expression	Low expression
Recurrent Metastatic lesions	Local recurrence	Positive	24(41.38)	10(17.24)	10(17.24)	12(20.69)	16(29.63)	13(24.07)	41 (70.69)	5 (8.62)
		Negative	17(29.31)	7 (12.07)	24(41.38)	12(20.69)	4 (7.41)	21(38.89)	10 (17.24)	2 (3.45)
	Distant metastasis	Positive	22(30.56)	19(26.39)	10(13.89)	14(19.44)	13(19.40)	11(16.42)	41 (56.94)	14 (19.44)
		Negative	21(29.17)	10(13.89)	24(33.33)	24(33.33)	6 (8.96)	37(55.22)	10 (13.89)	7 (9.72)

#### 3.2.2 Expression heterogeneity of PR between primary and metastatic breast cancer lesions

The PR expression was inconsistent between the primary and the recurrence/metastasis lesions in 67 cases, of which 41 (31.54%) changed from positive to negative and 26 (20.00%) changed from negative to positive. The primary lesion was PR- negative in 52 cases and changed to PR-positive in 26 (50.00%) cases. The primary lesion was PR-positive in 78 cases and changed to PR-negative in 41 (52.56%) cases. Among the patients with local recurrence, PR-positive turned negative in 24 cases, and negative turned positive in 12 cases, with an inconsistent expression rate of 62.07%. Among patients with distant metastasis, PR-positive turned negative in 24 cases, and negative turned positive in 14 cases, with an inconsistent expression rate of 52.78% (**Table 2**).

#### 3.2.3 Expression heterogeneity of HER2 between primary and metastatic breast cancer lesions

HER2 expression was inconsistent between the primary and the recurrence/metastasis lesions in 34 cases, of which 10 (8.26%) cases changed from positive to negative, and 24 (19.83%) cases changed from negative to positive. The primary lesion was HER2-negative in 82 cases and changed to HER2-positive in 24 (29.27%) cases. The primary lesion was HER2-positive in 39 cases and changed to HER2-negative in 10 (25.64%) cases. Among the patients with local recurrence, HER2-positive turned negative in 4 cases, and negative turned positive in 13 cases, with an inconsistent expression rate of 31.48%. Among patients with distant metastasis, HER2-positive turned negative in 6 cases and negative turned positive in 11 cases, with an inconsistent expression rate of 25.37% (**Table 2**).

#### 3.2.4 Expression heterogeneity of Ki-67 between primary and metastatic breast cancer lesions

Ki-67 expression was inconsistent between primary and recurrence/metastasis lesions in 38 cases, of which 19 (14.62%) cases changed from high to low expression, and 19 (14.62%) cases changed from low to high expression. Ki-67 expression was low in 29 cases of primary lesions and changed to high in 19 (65.52%) cases, while it was high in 101 cases of primary lesions and changed to high in 19 (18.81%) cases. Among patients with local recurrence, Ki-67 expression was changed from high to low in 10 cases and from low to high in 5 cases, with an inconsistent expression rate of 25.86%. Among patients with distant metastasis, Ki-67 expression was changed from high to low in 10 cases and from low to high in 14 cases, with an inconsistent expression rate of 33.33%.

### 3.3 Correlation between ER expression heterogeneity and DFS in patients

**Figure 1:**
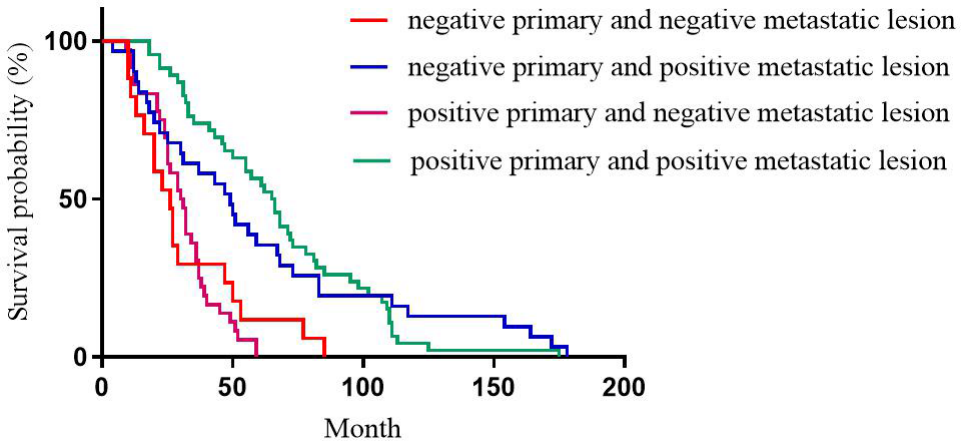
Correlation between ER expression heterogeneity and patient DFS.

For ER expression, the median DFS of patients with positive primary and positive metastatic lesion, positive primary and negative metastatic lesion, negative primary and positive metastatic lesion, negative primary and negative metastatic lesion was 66, 30, 55, and 27 months, respectively; the differences were statistically significant (p<0.001, **Figure 1**).

### 3.4 Correlation between PR expression heterogeneity and DFS in patients

**Figure 2:**
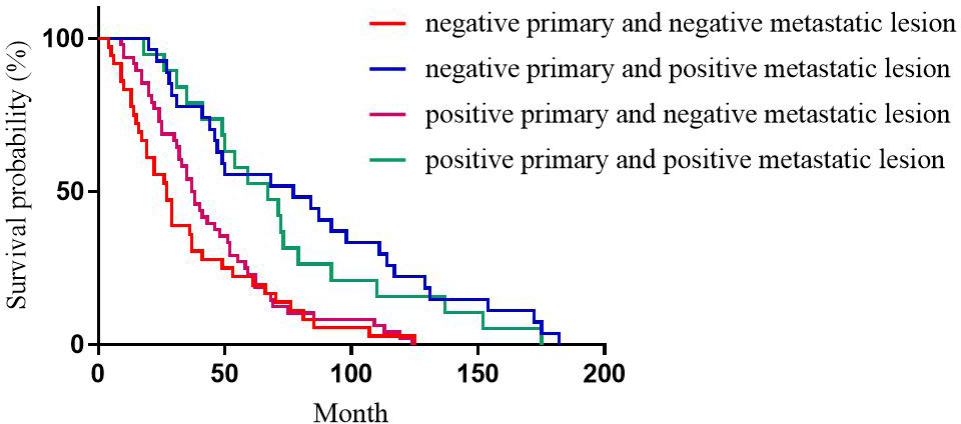
Correlation between PR expression heterogeneity and patient DFS.

For PR expression, the median DFS of patients with positive primary and positive metastatic lesion, positive primary and negative metastatic lesion, negative primary and positive metastatic lesion, negative primary and negative metastatic lesion was 67, 37, 77, and 27 months, respectively with statistically significant differences (p<0.001, **Figure 2**).

### 3.5 Correlation between HER2 expression heterogeneity and DFS in patients

**Figure 3:**
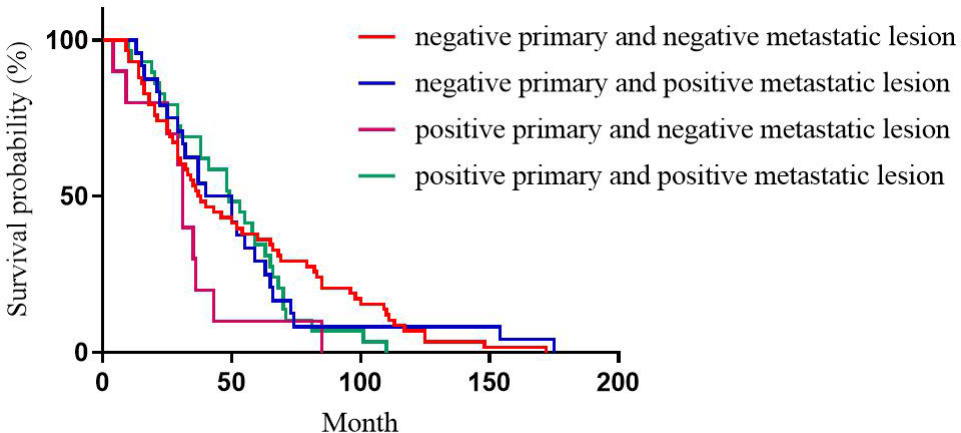
Correlation between HER2 expression heterogeneity and patient DFS.

For HER2 expression, the median DFS of patients with positive primary and positive metastatic lesion, positive primary and negative metastatic lesion, negative primary and positive metastatic lesion, negative primary and negative metastatic lesion was 49, 31, 40, and 38 months, respectively, with statistically significant differences (p=0.196, **Figure 3**).

### 3.6 Correlation between Ki-67 expression heterogeneity and DFS in patients

**Figure 4:**
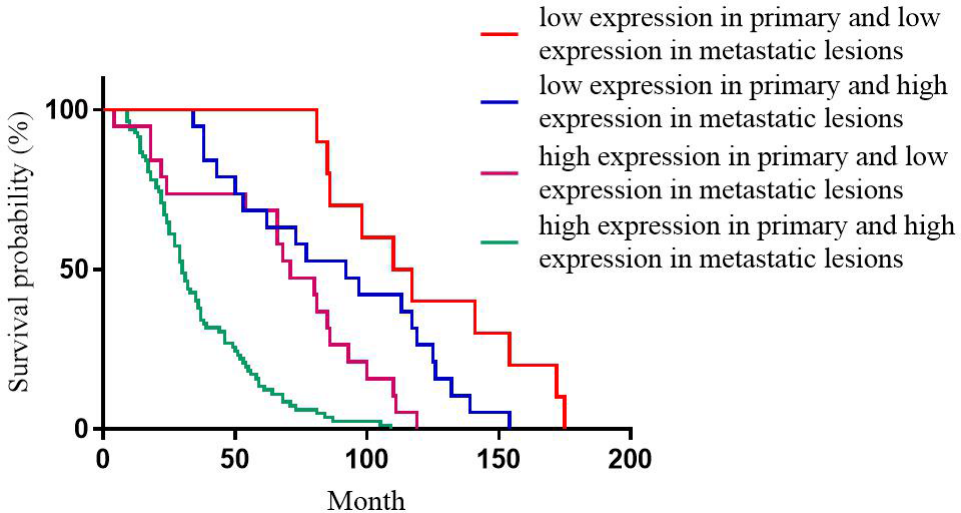
Correlation between the heterogeneity of Ki-67 expression and the DFS of patients.

For Ki-67 expression, the median DFS of patients with high expression in primary and high expression in metastatic lesions, high expression in primary and low expression in metastatic lesions, low expression in primary and high expression in metastatic lesions, low expression in primary and low expression in metastatic lesions was 30, 71, 97, and 110 months, respectively; the differences were statistically significant (p<0.001, **Figure 4**).

### 3.7 Correlation between tumor size, lymph node metastasis, and receptor expression heterogeneity

A total of 108, 108, 106, and 108 cases with complete TNM staging records of primary lesions corresponded to ER, PR, HER2, and Ki-67, respectively. The results of this study showed that tumor size did not correlate with the changes in the expression of ER, PR, HER2, and Ki-67 (p=0.208, 0.068, 0.823, and 0.781, respectively). The expression of ER, PR, HER2, and Ki-67 was correlated with the primary lesion accompanied by lymphatic metastasis (p=0.046, 0.036, 0.030, and 0.027, respectively) (**Table 3**).

**Table 3:** Correlation between T, N, and the expression changes of immunohistochemical indexes.

		Change in ER		Change in PR		Change in HER2		Change in Ki-67	
		YES	NO	YES	NO	YES	NO	YES	NO
T	T1	14	12	17	10	10	16	7	23
	T2	22	31	31	22	14	32	15	34
	T3	13	8	10	9	5	14	7	13
	T4	2	6	2	7	5	9	2	7
	P	0.208		0.068		0.823		0.781	
N	N0	15	27	29	13	8	34	18	24
	N1-N3	35	28	32	34	25	39	15	51
	P	0.046		0.036		0.03		0.027	

### 3.8 Effect of treatment regimen on receptor expression heterogeneity

Primary breast cancer treatment included anthracycline chemotherapy, paclitaxel chemotherapy, endocrine therapy, and targeted therapy in 92, 87, 82, and 39 cases, respectively. Logistic regression analysis showed that anthracycline chemotherapy, paclitaxel chemotherapy, endocrine therapy, and targeted therapy did not affect the differences in the expression of ER, PR, HER2, and Ki-67 between primary and metastatic lesions (**Tables 4**–**7**, respectively).

**Table 4:** Logistic multivariate analysis results of treatment regimens on changes in ER expression.

Therapy regimen	B	standard error	Wald value	OR	95% CI	P
Targeted therapy	-0.602	0.461	1.704	0.548	0.222-1.352	0.192
Endocrine therapy	1.208	0.972	1.544	3.346	0.498-22.485	0.214
Taxane-containing chemotherapy	-0.405	1.354	0.090	0.667	0.047-9.472	0.765
Anthracycline-containing chemotherapy	0.693	1.056	0.431	2.000	0.252-15.489	0.512

**Table 5:** Logistic multivariate analysis results of treatment regimens on changes in PR expression.

Therapy regimen	B	Standard error	Wald value	OR	95% CI	P
Targeted therapy	-0.682	0.462	2.18	0.506	0.204-1.25	0.140
Endocrine therapy	1.242	0.971	1.634	3.462	0.516-23.233	0.201
Taxane-containing chemotherapy	-0.405	1.354	0.090	0.667	0.047-9.472	0.765
Anthracycline-containing chemotherapy	1.355	1.076	1.585	3.875	0.471-31.912	0.208

**Table 6:** Logistic multivariate analysis results of treatment regimens on changes in HER2 expression.

Therapy regimen	B	Standard error	Wald value	OR	95% CI	P
Targeted therapy	-0.178	0.548	0.105	0.837	0.286-2.449	0.746
Endocrine therapy	-0.195	0.795	0.060	0.823	0.173-3.908	0.806
Taxane-containing chemotherapy	1.232	0.972	1.607	3.429	0.510-23.047	0.205
Anthracycline-containing chemotherapy	-0.813	1.075	0.572	0.443	0.054-3.645	0.449

**Table 7:** Logistic multivariate analysis results of treatment regimens on changes in Ki-67 expression.

Therapy regimen	B	Standard error	Wald value	OR	95% CI	P
Targeted therapy	0.875	0.49	3.194	2.400	0.919-6.269	0.074
Endocrine therapy	-1.281	1.138	1.266	0.278	0.030-2.586	0.260
Taxane-containing chemotherapy	0.916	1.396	0.431	2.500	0.162-38.599	0.512
Anthracycline-containing chemotherapy	-0.56	0.954	0.344	0.571	0.088-3.709	0.558

## 4 Discussion

The ER, PR, and HER2 expression status and the level of Ki-67 were critical indicators for formulating treatment strategies for breast cancer patients. According to the varied expression status, the receptors closely related to the treatment and prognosis of breast cancer patients can be divided into different subtypes. For patients with advanced breast cancer, previous treatment is based on the receptor status of the patient’s primary lesion. In recent years, retrospective studies have shown significant differences in the expression of ER, PR, and HER2 receptors between primary and metastatic breast cancer lesions. The generation of receptor expression heterogeneity may be related to the heterogeneity and the polyclonal nature of tumor tissues, clonal selection of tumor cells, tissue fixation, antigen repair, differences in staining methods, pathologists’ subjective judgment of staining results, and previous treatment (6–9) A meta-analysis showed that ER was 3-54% inconsistent, PR 5-78%, and HER2 0-34% inconsistent (10). In this study, the inconsistent rates of ER, PR, HER2, and Ki-67 were 47.69%, 51.54%, 28.10%, and 29.23%, respectively. This difference in receptor expression might influence the choice of treatment for patients with metastatic breast cancer (11), and that in receptor expression might influence the treatment choice for patients with metastatic breast cancer. The loss of receptor positivity in metastatic lesions may result in ineffective treatment and adverse drug reactions, while a lack of understanding of the receptor positivity status in metastatic lesions might lead to incorrect treatment: both had an impact on patient survival expectations. The majority of the studies showed that ER, PR, and HER2 receptors were changed from positive to negative, and the expression of Ki-67 was changed from low to high (12, 13), while some studies showed that the conversion rate of ER, PR, and HER2 receptors to positive was higher than that of negative (14, 15).

Some studies found that the inconsistency rate of ER, PR, and HER2 expression in distant metastatic lesions is higher than that in local recurrent lesions, which could be attributed to the fact that local recurrent lesions are formed by the proliferation of primary tumor cells that have not been cleared and are likely to maintain their original receptor expression (6, 16). However, distant metastatic lesions are formed by the distal colonization and proliferation of tumor cells through lymphatic vessels or blood vessels and are likely to express receptors different from those in the primary lesions. In the present study, the inconsistent expression rates of ER, PR, HER2, and Ki-67 in patients with local recurrence were 46.55%, 62.07%, 31.48%, and 25.86%, respectively, while the inconsistent expression rates of ER, PR, HER2, and Ki-67 in patients with distant metastasis were 55.56%, 52.78%, 25.37%, and 33.33%, respectively. Moreover, no statistically significant differences were observed in the expression of ER, PR, HER2, and Ki-67 in the primary breast cancer foci and recurrence and metastasis foci; the total change rates of ER, PR, HER2, and Ki-67 were 47.69%, 51.54%, 28.10%, and 29.23%, respectively. However, a large difference in the receptor expression between primary and metastatic lesions severely affected the development of treatment regimens and the assessment of patient outcomes. The mechanism of expression changes in ER, PR, and HER2 between primary breast cancer lesions and their recurrence/metastasis is unclear. It might be related to gene drift, tumor evolution, tumor heterogeneity, and treatment plan. The expression of the Ki-67 protein reflects the activity of tumor cells and is highly correlated with the development, metastasis, and prognosis of malignant tumors (17, 18). Notably, 17 cases of triple-negative breast cancer were included in this study, among which 3 were transformed into the luminal type, and 2 were transformed into the HER2 amplified type. Endocrine or targeted therapy could improve the outcomes after receptor expression shifts in metastatic lesions. Therefore, it could be speculated that re-biopsy of both locally recurrent and distant metastatic lesions should be actively performed to determine receptor expression and avoid missing endocrine or targeted therapy for new receptor expression.

This study showed that for ER expression, the median DFS of patients with positive primary and positive metastatic lesion, positive primary and negative metastatic lesion, negative primary and positive metastatic lesion, negative primary and negative metastatic lesion was 65, 30, 49, and 65 months, respectively, with statistically significant differences (p<0.001). Among these cases, patients with positive ER expression in both primary and metastatic lesions had the longest DFS, and patients with negative ER expression in both primary and metastatic lesions had the shortest DFS, which was consistent with the previous studies (12, 17, 19). This phenomenon could be attributed to ER as an independent indicator to judge the prognosis of breast cancer (20), and the expression level of ER is closely related to the prognosis and DFS of breast cancer patients. Therefore, treatment strategies should be adjusted after ER expression turns negative, such as after starting chemotherapy. Subsequently, after ER expression changes from negative to positive, patients should be given the opportunity of endocrine therapy for prolonged survival. This study showed that for PR expression, the median DFS of patients with positive primary and positive metastatic lesion, positive primary and negative metastatic lesion, negative primary and positive metastatic lesion, negative primary and negative metastatic lesion was 67, 37, 77, and 27 months, respectively, with statistically significant differences (p<0.001). In patients with breast cancer, the comparison of immunohistochemical results between primary and metastatic lesions revealed that ER levels decreased slightly after endocrine therapy, while PR levels decreased dramatically, with up to half of the tumors completely losing PR expression with the development of drug resistance (21). Therefore, the loss of PR in metastatic lesions might be a major marker of failure to respond to endocrine therapy (9, 22). Meng et al. compared the survival of breast cancer patients with altered PR expression from negative to positive and showed better survival than for PR changed from positive to negative (23). For ER and PR, the survival was significantly reduced in patients with a positive primary and a negative metastatic lesion compared to a positive primary and a positive metastatic lesion. Conversely, no significant difference was observed in the survival of patients with negative primary and positive metastatic lesions compared to patients with negative primary and negative metastatic lesions. However, there is no consensus on the effect of receptor transformation on survival (24, 25). This phenomenon could be attributed to PR as an ER-dependent gene product, and PR synthesis requires a complete ER-PR pathway. Theoretically, PR expression is an indicator of the functional integrity of the ER pathway, such that the expression of PR can accurately evaluate the efficacy of endocrine therapy. However, about 1-4% of patients with positive expression of PR after ER deletion indicated that some breast cancer PR expression was independent of ER expression (26). This prognostic effect of ER on breast cancer has been proven previously, but the role of PR has been controversial. While evaluating the prognosis of patients, changes in PR expression should be combined with changes in ER expression. This study showed that for HER2 expression, the median DFS of patients with positive primary and positive metastatic lesion, positive primary and negative metastatic lesion, negative primary and positive metastatic lesion, negative primary and negative metastatic lesion was 49, 31, 40, and 37 months, respectively, with no statistical significance (p=0.218). Some studies have shown that when the expression of receptors is inconsistent between metastatic and primary foci, the prognosis of patients with altered HER2 expression is worse than that of patients with unchanged HER2 expression, possibly because patients with altered HER2 expression did not receive appropriate anti-HER2 therapy (24). Other studies have shown that patients with HER2-positive metastatic lesions who received trastuzumab had a better prognosis than patients with HER2-negative metastatic lesions and patients with HER2-positive metastatic lesions who did not receive trastuzumab (27).We also observed that for Ki-67 expression, the median DFS of patients with high expression in primary and high expression in metastatic lesions, high expression in primary and low expression in metastatic lesions, low expression in primary and high expression in metastatic lesions, low expression in primary and low expression in metastatic lesions was 30, 71, 97, and 110 months, respectively, with statistically significant differences (p<0.001). Patients with low Ki-67 expression in both primary and metastatic foci had the longest DFS and the best prognosis, while those with high Ki-67 expression in both primary and metastatic foci had the shortest DFS and worst prognosis. This could be because Ki-67, a DNA-binding protein, is overexpressed in various malignant tumor diseases (28, 29), reflecting tumor cell activity, and is highly correlated with the development, metastasis, and prognosis of malignant tumors (30, 31). Several studies have shown that patients with a high expression level of Ki-67 antigen are prone to breast cancer lymph node metastasis. The later the disease stage of patients with recurrent metastatic breast cancer, the higher the positive rate of Ki-67 expression in cancer tissues. Breast cancer with a high expression of Ki-67 is highly invasive, and breast cancer cells are prone to recurrence and metastasis (31).

Tumor heterogeneity may be influenced by the tumor microenvironment, irrespective of whether the primary tumor is associated with lymph node metastasis and the treatment of the primary lesion. A previous study has shown that tumor microenvironment and treatment plan promote tumor heterogeneity (32). In this study, the tumor size was not associated with the altered expression of ER, PR, HER2, and Ki-67 (p=0.208, 0.068, 0.823, and 0.781, respectively). We also showed that the altered expression of ER, PR, HER2, and Ki-67 was related to whether the primary lesion was accompanied by lymphatic metastasis (p=0.046, 0.036, 0.030, and 0.027). This phenomenon may be because the primary tumor cells with lymphatic metastasis are aggressive, and the tumor is prone to metastasis and receptor changes. Reportedly, the inconsistent expression of receptors in primary and metastatic lesions may be related to the choice of treatment regimen (33). Conversely, some studies have shown that the correlation between the two parameters is not statistically significant (34). In the current study, anthracycline, paclitaxel, endocrine therapy, and targeted therapies were not associated with receptor transformation between primary and metastatic lesions. Presently, the effect of treatment regimens on receptor changes between primary and metastatic lesions is controversial, necessitating an in-depth investigation.

This was a single-center retrospective study with a small number of cases dependent on the pathological reports to obtain receptor expression information. Moreover, the biopsy of the metastatic lesions was carried out by puncture biopsy. Due to tumor heterogeneity, insufficient puncture tissue and different puncture sites may produce different results. Moreover, FISH could not be performed on patients with HER2 (++) due to limited sampling and patient willingness.

In conclusion, after the recurrence and metastasis of breast cancer, the expressions of ER, PR, HER2, and Ki-67 may be inconsistent between the primary and metastatic lesions, which is significant for the formulation of follow-up treatment plans and the evaluation of prognosis. Therefore, in a clinical setting, re-biopsy should be performed for patients with recurrent and metastatic breast cancer every time the disease progresses to assess the changes in molecular phenotype and strive for individualized and precise treatment for patients.

## Data availability statement

The original contributions presented in the study are included in the article/supplementary material. Further inquiries can be directed to the corresponding author.

## Author contributions

XH, FL, and KG designed the study. XH, WC, FL, PR, YW, and CZ collected the data. XH, WC, and PR analyzed the data and drafted the manuscript. KG is the corresponding author who instructed the research. All authors contributed to the article and approved the submitted version.
